# Accuracy of cervical auscultation in detecting the presence of material in the airway

**DOI:** 10.1002/cre2.89

**Published:** 2017-11-16

**Authors:** Shinji Nozue, Yoshiaki Ihara, Koji Takahashi, Yuka Harada, Yoshiko Takei, Ken Yuasa, Kaoru Yokoyama

**Affiliations:** ^1^ Division of Oral Rehabilitation Medicine, Department of Special Needs Dentistry School of Dentistry, Showa University Tokyo Japan

**Keywords:** cervical auscultation, dysphagia, penetration‐aspiration scale, sensitivity, specificity, videofluorographic swallowing study

## Abstract

Several studies have investigated the accuracy of cervical auscultation (CA). However, both the sensitivities and the specificities of CA in detecting dysphagic conditions varied widely among these studies. These wide variations of the accuracy of CA might be caused by differences of the targeted sounds, such as the expiratory sound (ES) and/or swallowing sound (SS). Forty‐six dysphagic patients were served as subjects. Patients who had unoccluded tracheostoma and patients who could not follow the instructions were excluded. During the videofluorographic swallowing study (VFSS), the subjects swallowed 3 ml of yogurt containing barium sulfate. The VFSS images were recorded with acoustic signals including both the swallowing and respiratory sounds detected by our method. Classification of the VFSS images was decided by consensus of the three dentists using a penetration‐aspiration scale (PAS). Recorded VFSS images were classified into the following 2 groups based on PAS: “no or minimum risk group”: PAS1–2; and “possible risk group”: PAS3–8. As a result of the classification of VFSS findings, 30 samples were evaluated as no or minimum risk group and 16 as possible risk group. Twelve observers including 10 dentists other than 3 dentists who evaluated VFSS images and 2 speech pathologists auditorily diagnosed “negative” and “positive.” Sensitivity, specificity, and intra‐rater reliability was calculated for the 3 types of acoustic samples. The sensitivity of the intra‐rater reliability was 60.3% for ES, 76.6% for SS, and 89.9% for ES + SS. The sensitivity of intra‐rater reliability of ES + SS samples was significantly higher than that of ES (p < .01) and SS (p < .05). The sensitivity of intra‐rater reliability of SS was significantly higher than that of ES (p < .01). The specificity of the intra‐rater reliability was 53.7% for ES, 50.3% for SS, and 44.5% for ES + SS. ES + SS might be most useful for detecting the presence of material in the airway.

## 1 INTRODUCTION

Videofluorographic swallowing study (VFSS) and fiberoptic endoscopic examination of swallowing (FEES) are widely used in the field of dysphagia management. Both of these examinations are very effective in diagnosing dysphagia. VFSS images shows oral, pharyngeal, and cervical‐esophageal bolus flow during swallowing. Anatomic and/or physiologic abnormalities are identified relative to swallowing. However, VFSS is an invasive examination with radiation exposure to the patients through fluoroscopic procedures. Furthermore, the taste, smell, and texture of test food or liquid materials used in VFSS are different from those original, because test materials contain radiopaque agent such as barium sulfate or iodine‐based contrast agent. On the contrary, daily foods and liquids can be used in FEES. FEES allows inspection of functions of the swallowing mechanism at the velopharynx, oropharynx, pharynx, and larynx. However, it does not permit any systematic evaluation of oral or esophageal components of swallowing. During FEES, passage of the bolus and movement of the structures cannot be observed at the moment of the swallow because tissue surrounds the end of the endoscopy, causing a brief condition referred to as “white‐out.” Furthermore, FEES is also invasive because uncomfortable sensation is given to the patient during nasal endoscopic procedures (Martin‐Harris & Jones, 2008; Nacci et al., 2008; Wilson & Howe, 2012).

Cervical auscultation (CA) is a portable, non‐invasive technique that uses a stethoscope to detect cervical sounds generated during the swallow and breath sounds pre‐ and post‐swallow. CA is widely used for estimating dysphagic conditions such as aspiration, penetration, and pharyngeal retention in the various clinical settings (Takahashi, Groher, & Michi, 1994a; Takahashi, Groher, & Michi, 1994b; Uyama, Takahashi, Michi, & Kawabata, 1997). Several studies focusing on the investigation of the accuracy of CA in detecting dysphagic conditions of the pharynx and the larynx have been reported. However, both the sensitivities and the specificities of CA in detecting dysphagic conditions varied widely among these studies; a sensitivity varying from 23% to 94%, and a specificity varying from 50% to 74% (Lagarde, Kamalski, & van den Engel‐Hoek, 2016). One possible reason for these wide variations among the CA studies might be caused by the differences of targeted sounds. Some studies focused on expiratory sounds (ES) pre‐ and post‐swallow (Hirano, Takahashi, Uyama, & Michi, 2001; Zenner, Losinski, & Mills, 1995), whereas others focused on swallowing sounds (SS) alone (Bergström, Svensson, & Hartelius, 2014; Borr, Hielscher, & Lücking, 2007; Leslie, Drinnan, Finn, Ford, & Wilson, 2004; Santamato et al., 2009; Stroud, Lawrie, & Wiles, 2002). Therefore, the sensitivity and the specificity of CA using both swallowing and respiratory sounds for detecting dysphagic conditions are still unclear. In this study, we investigated the specificity and sensitivity of CA using three types of acoustic samples: pre‐ and post‐swallow ES, SS alone, and SS with pre‐ and post‐swallow ES (ES + SS) in the detection of dysphagia.

## 2 METHODS

### 2.1 Subjects

Forty‐six mixed dysphagic adult patients including 21 post‐surgical head and neck cancer patients, 11 cerebral disease patients, 5 psychogenic dysphagia patients, 5 aged sarcopenia patients without any disease, 3 esophageal cancer patients, and 1 amyotrophic lateral sclerosis patient were served as subjects. All subjects were able to follow our instructions and received VFSS from April 2011 to July 2013 at Showa University Dental Hospital. The participant characteristics are presented in Table 1. Patients who had unoccluded tracheostoma and patients who could not follow the instructions were excluded. Patients with fatigue, fever, and/or any other poor physical conditions that might influence on swallow function were also excluded.

**Table 1:** Participant characteristics

Age	Gender	Diagnosis	PAS
44	Male	Tongue cancer	1
67	Male	Tongue cancer	3
72	Male	Tongue cancer	3
68	Male	Tongue cancer	4
80	Male	Tongue cancer	5
89	Male	Tongue cancer	5
78	Male	Oropharyngeal cancer	1
85	Male	Oropharyngeal cancer	2
39	Female	Oropharyngeal cancer	8
66	Male	Oropharyngeal cancer	8
85	Male	Hypopharyngeal cancer	1
73	Male	Hypopharyngeal cancer	2
85	Male	Hypopharyngeal cancer	2
73	Male	Hypopharyngeal cancer	5
83	Female	Thyroid cancer	2
63	Male	Thyroid cancer	5
63	Male	Thyroid cancer	6
85	Male	Carcinoma of mandibule	2
85	Male	Carcinoma of mandibule	8
69	Male	Epipharynx cancer	2
55	Male	Carcinoma of floor of mouth	8
52	Female	stroke	1
79	Female	stroke	1
83	Female	stroke	1
65	Male	stroke	2
86	Male	stroke	2
71	Female	Cerebral tumor	1
71	Female	Cerebral tumor	5
85	Male	Alzheimer dementia	6
89	Female	Craniocerebral trauma	5
64	Female	Epilepsy	1
57	Male	Hypoxic encephalopathia	2
80	Male	Sarcopenia	1
83	Male	Sarcopenia	1
83	Male	Sarcopenia	1
83	Male	Sarcopenia	1
86	Male	Sarcopenia	1
40	Female	psychogenic dysphagia	1
60	Male	psychogenic dysphagia	1
78	Male	psychogenic dysphagia	1
82	Female	psychogenic dysphagia	2
82	Female	Depression	1
72	Male	Esophageal cancer	2
72	Male	Esophageal cancer	2
73	Male	Esophageal cancer	4
76	Female	Amyotrophic lateral sclerosis	2

*Note*. PAS = penetration‐aspiration scale.

### 2.2 Detection and acquisition of voluntary elicited expiratory sound and swallowing sound samples

**Figure 1:**
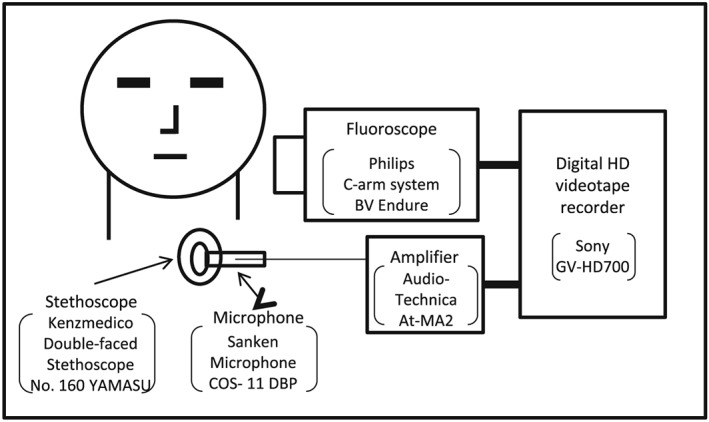
Schematic diagram of recording in the VFSS. The acoustic signals of pre/post‐swallowing expiratory sounds and swallowing sounds were amplified, digitally converted at a sampling rate of 48 kHz, and recorded with VFSS images on to DVCAM tape through a digital high‐definition videotape recorder

The diaphragm chest piece of a double‐faced stethoscope connected to a short tube with an inserted microphone was attached to the site over the lateral border of the trachea immediately inferior to the cricoid cartilage using a 1 cm^2^ piece of double‐sided adhesive paper tape (Takahashi et al., 1994a). The clearing procedure for discharging residues was done in the patient with suspected oral and/or pharyngeal and/or laryngeal residues. Before VFSS, oral and/or pharyngeal and/or laryngeal residues were cleared by strong voluntary coughing or forced expiration with keeping a forward‐bent posture. When clearing oral and/or pharyngeal and/or laryngeal residues was not verified after repeated clearing procedures, suctioning was required for clearing residues. After clearance of the airway was confirmed, the patient was asked to practice exhaling three times with constant force. During the VFSS, the subjects swallowed 3 ml of yogurt containing barium sulfate adjusted 1:1 as the weight ratio. The yogurt was injected into the mouth using a syringe. Just after swallowing the yogurt, the subjects were told to exhale voluntarily three times with constant force. The sequentially detected acoustic signals including pre‐swallow ES, SS, and post‐swallow ES were amplified, digitally converted at a sampling rate of 48 kHz and recorded with VFSS images on DVCAM tape using a digital high‐definition videotape recorder. A diagram of the recording system is presented in Figure 1 (Yamashita et al., 2014).

### 2.3 Grouping of samples according to penetration‐aspiration scale of VFSS findings

Grouping of samples was performed according to the Rosenbek's penetration‐aspiration scale (PAS) of VFSS findings: Acceptable swallow (no or minimum risk group [NM]: PAS1, 2), and not acceptable dysphagic swallow (possible risk group [P]: PAS 3–8; Rosenbek, Robbins, Roecker, Coyle, & Wood, 1996; Landis & Koch, 1977).

Grading of PAS of all VFSS findings was decided by reaching consensus among three dentists who had more than 5 years of clinical experience in the dysphagia management.

### 2.4 Editing acoustic samples and discriminating edited sounds by auditorily evaluation

The sequentially detected acoustic signals during VFSS including pre‐swallow ES, SS, and post‐swallow ES were edited to three categories of sounds using edius for Windows (edius pro6.5). Each of three edited categories of the sequentially detected acoustic signals is as follows. Pre‐swallow ES and post‐swallow ES were edited as pre‐ and post‐swallow ES. SS were edited as SS alone. The sequentially detected pre‐swallow ES, SS, and post‐swallow ES were edited as sequential pre‐swallow expiratory, swallowing, and post‐swallow ES (ES + SS). Twelve raters listened to all edited sound samples through an open headphone. Each of three categories of the edited sounds was presented to each rater once in the order of ES, SS, and ES + SS. Presentation of each category of the edited sounds to each rater was performed in 1 week of interval. The raters evaluated auditorily each edited sound and discriminated it “negative” or “positive.”

**Figure 2:**
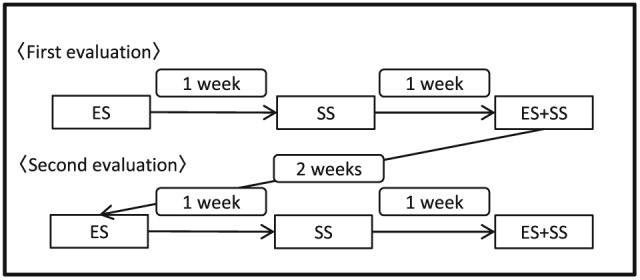
Schedule of discrimination examination. The first evaluation was performed in the order of expiratory sound (ES), swallowing sounds (SS), and ES + SS. There was 1 week spacing between each discrimination examination. The second evaluation was performed 2 weeks after the first evaluation

A series of these auditory evaluation protocol was carried out twice in 2 weeks of interval (Figure 2).

### 2.5 Statistical analysis

The percentage of correctly diagnosed positive samples with matching results of both the VFSS findings and auditory evaluations was calculated to determine the sensitivity. The percentage of correctly diagnosed negative samples with matching results of the two experiments was also calculated to determine the specificity.

The sensitivities and the specificities of the three types of acoustic sounds were compared using Scheffe's test. We calculated intra‐rater reliability using kappa scores. The benchmarks provided by Landis and Koch to rate kappa values on a scale of “poor” to “almost perfect,” although familiar and popularly used and can be over simplistic if regarded as universally applicable (Landis & Koch, 1977). The percentages of correctly diagnosed positive and negative samples were calculated for detecting agreement in the two sets of examination to find the sensitivity and specificity in intra‐rater reliability. The sensitivity, specificity, and intra‐rater reliability of the three types of acoustic sounds were compared using Scheffe's test. Results were accepted as statistically significant at the 5% level of probability. Data were analyzed with spss for Windows (ibm spss Statistics 20).

The ethics committee of Showa University School of Dentistry granted approval for this study (no. 2014‐018).

## 3 RESULTS

### 3.1 Classification from videofluorography images

Three dentists classified samples according to the PA scale. As a result, 30 samples were evaluated as NM, and 16 samples were P. In the NM group, 17 samples were PAS 1 and 13 samples were PAS 2. In the P group, two samples were PAS 3, two samples were PAS 4, six samples were PAS 5, two samples were PAS 6, and four samples were PAS 8.

### 3.2 Sensitivity and Specificity

For ES samples, the sensitivity at the first evaluation was 57.6% and that at the second evaluation was 59.9%. The specificity at the first evaluation was 54.3% and that at the second evaluation was 51.0%. For SS samples, sensitivity at the first evaluation was 72.3% and that at the second evaluation was 71.6%. The specificity at the first evaluation was 49.6% and that at the second evaluation was 51.7%. For ES + SS samples, sensitivity at the first evaluation was 81.2% and that at the second evaluation was 83.9%. The specificity at the first evaluation was 46.9% and that at the second evaluation was 44.1% (Table 2). The sensitivity at the second evaluation in ES and ES + SS was higher than that at the first evaluation. The sensitivity of the first evaluation in SS was higher than that of the second evaluation. However, there was no significant difference in the sensitivity (*p* > .05). The sensitivity of SS and ES + SS was significantly higher than that of ES at the first evaluation and second evaluation (*p* < .01). ES + SS sensitivity was greater than ES sensitivity. There was no significant difference at the first evaluation (*p* > .05). However, at the second evaluation, there was a significant difference (*p* < .05). The specificity of the second evaluation in SS was higher than that of the first evaluation. However, there was no significant difference in the specificity (*p* > .05). The specificity of ES was higher than that of SS and ES + SS at the first evaluation and second evaluation. However, there was no significant difference in the specificity of the results (*p* > .05). Specificity was low in all three types of acoustic samples.

**Table 2:** Sensitivity and specificity

	Cervical auscultation	
	Positive	Negative
P	sensitivity	false negative
NM	false positive	specificity
ES (First evaluation)		

	Cervical auscultation	
	Positive	Negative
P	57.6	42.4
NM	45.7	54.3
ES (Second evaluation)		

	Cervical auscultation	
	Positive	Negative
P	59.9	40.1
NM	49.0	51.0
SS (First evaluation)		

	Cervical auscultation	
	Positive	Negative
P	72.3	27.7
NM	50.4	49.6
SS (Second evaluation)		

	Cervical auscultation	
	Positive	Negative
P	71.6	28.4
NM	48.3	51.7
ES + SS (First evaluation)		

	Cervical auscultation	
	Positive	Negative
P	81.9	18.1
NM	53.1	46.9
ES + SS (Second evaluation)		

	Cervical auscultation	
	Positive	Negative
P	83.9	16.1
NM	55.1	44.1

*Note*. NM = no or minimum risk group; P = possible risk group; SS = swallowing sounds; ES = expiratory sound.

### 3.3 Intra‐rater reliability

The intra‐rater reliability of ES samples had 0.47 of kappa. It judges rated “moderate.” The intra‐rater reliability of SS samples had 0.64 of kappa. It judges rated “good.” The intra‐rater reliability of ES + SS samples had 0.60 of kappa. It judges rated good.

### 3.4 Intra‐rater reliability (sensitivity and specificity)

**Figure 3:**
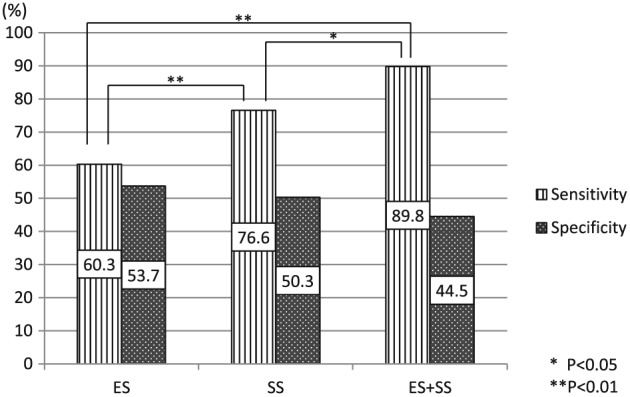
The intra‐rater reliability results (sensitivity and specificity). The sensitivity of the intra‐rater reliability of swallowing sounds (SS) was significantly higher than that of expiratory sound (ES) (**p < .01), and that of ES + SS was significant higher than that of SS (*p < .05) and that of ES (*p* < .01)

The sensitivity of the intra‐rater reliability of ES samples was 60.3%. The corresponding values in SS samples and ES + SS samples were 76.6% and 89.8%, respectively. The sensitivity of intra‐rater reliability of ES + SS samples was significantly higher than that of ES (*p* < .01) and SS (*p* < .05). The sensitivity of intra‐rater reliability of SS was significantly higher than that of ES (*p* < .01). The specificity of the intra‐rater reliability of ES samples was 53.7%. The corresponding values in SS samples and ES + SS samples were 50.3% and 44.5%, respectively. The specificity of the intra‐rater reliability of ES samples was the highest. However, there was no significant difference between the intra‐rater reliability of ES and SS and ES + SS (*p* > .05; Figure 3).

## 4 DISCUSSION

### 4.1 Accuracy of CA

In this study, we compared ES, SS, and ES + SS in each subjects. We calculated the sensitivity and specificity of CA. Furthermore, we calculated the intra‐rater reliability and the sensitivity and specificity of the intra‐rater reliability to evaluate the reliability of CA. ES + SS showed the highest sensitivity, intra‐rater reliability, and sensitivity of intra‐rater reliability compared with the other types of acoustic samples. We think that raters could grasp a state of the oropharyngeal by pre‐swallowing ES and could grasp a swallowing state by SS, could compare it with pre‐swallowing by post ES in these results. All three types of acoustic samples exhibited low specificity and specificity of intra‐rater reliability. This result meant that ES + SS had high sensitivity and low specificity. Same tendency (high sensitivity and low specificity) was recognized in some prior studies focused on other screening methods (Lim et al., 2001; Tohara, Saitoh, Mays, Kuhlemeier, & Palmer, 2003). This tendency means that it will detect aspirator patients with high probability; on the other hand, it will detect many no aspirator patients as aspirator in spite they are healthy. In general, screening tests carried out prior to a detailed examination (VFSS or FEES). Therefore, screening tests for abnormal are inquired that it will detect all patients who need a detailed examination. In contraction, if the screening tests have low sensitivity and high specificity, it means the missing a case of aspiration, and it might be increase the risk of pneumonia. The result of misidentify no aspirator patients as aspirator are less serious than the reverse. Therefore, these results suggest that ES + SS would be the most useful sound sample to detect penetration and aspiration.

In the studies by Borr, Leslie, Zenner, Santamato, and Hirano, the specificity of CA was high. The high specificity reported in these studies could be explained by two factors. First, the studies used healthy subjects, and second, the quantity of food material swallowed. Studies by Borr, Leslie, and Santamato were performed in healthy subjects (Borr et al., 2007; Leslie et al., 2004; Santamato et al., 2009), while in the studies by Zenner and Hirano, the subjects were dysphagic patients and not healthy subjects (Hirano et al., 2001; Zenner et al., 1995). The subjects of this study were not healthy subjects. Their SS might be weaker and shorter compared with healthy subjects. And we used 3 ml of yogurt containing barium sulfate based on a modified water swallowing test; however, other studies used over 5 ml of material, which would require more time for transmission through the oropharynx and hypopharynx than when using less material. When swallowing a smaller quantity of material, the swallowing time was increased and the raters listened to short SS (Sdravou, Walshe, & Dagdilelis, 2012). Therefore, the raters might have judged the samples as positive.

The ideal screening test provides both the highest sensitivity and specificity. However, there were many screening tests that provided high sensitivity and low specificity or low sensitivity and high specificity. In the various clinical settings, a dysphagia screening test with low sensitivity detects positive in the low probability. In this study, ES + SS indicated the highest sensitivity and the lowest specificity in three types of acoustic samples. We judged that ES + SS was the most useful acoustic sample in CA. As the aim of a screening test is to detect an abnormal swallow, the screening test must have high sensitivity, but there is relatively little need for specificity (Kagaya et al., 2010; Tohara et al., 2003).

### 4.2 Limitations

In this study, we evaluated three types of acoustic samples in patients who could follow our instructions. However, there are many patients who were unable to follow instructions because of their disease or level of consciousness at the hospital or nursing home. Therefore, it is necessary to identify a method of CA that is suitable for such patients. We used 3 ml of yogurt containing barium sulfate only. Future investigations using other materials are necessary. In this study, because the raters judged the sample as positive, when a small amount of material was swallowed, it is necessary to conduct further examinations with varying quantities of material (Hammoudi, Boiron, Hernandez, Bobillier, & Morinière, 2014; Youmans & Stierwalt, 2011).

## 5 CONCLUSION

We investigated the sensitivity and specificity of CA using three types of acoustic samples: ES, SS, and ES + SS. We compared these three types of acoustic samples in terms of sensitivity, specificity, intra‐rater reliability, and sensitivity and specificity of intra‐rater reliability. ES + SS showed the highest sensitivity, intra‐rater reliability and sensitivity of intra‐rater reliability than other types of acoustic samples. However, all three types of acoustic samples exhibited low specificity and specificity in intra‐rater reliability. These results suggested that ES + SS is useful to detect abnormal swallow and is a reproducible method. Because the aim of a screening test is to detect an abnormal swallow, the screening test must have high sensitivity and relatively little importance for specificity.

## CONFLICT OF INTEREST

None of the authors have any conflict of interests to declare.
